# Antibacterial activity of tannins isolated from *Sapium baccatum* extract and use for control of tomato bacterial wilt

**DOI:** 10.1371/journal.pone.0181499

**Published:** 2017-07-25

**Authors:** Thuy Thu Vu, Hun Kim, Vu Khac Tran, Hoang Dinh Vu, Tien Xuan Hoang, Jae Woo Han, Yong Ho Choi, Kyoung Soo Jang, Gyung Ja Choi, Jin-Cheol Kim

**Affiliations:** 1 Department of Green Chemistry and Environmental Biotechnology, Korea University of Science and Technology, Daejeon, Republic of Korea; 2 Center for Eco-friendly New Materials, Korea Research Institute of Chemical Technology, Daejeon, Republic of Korea; 3 Department of Pharmaceutical Chemistry and Pesticides Technology, Hanoi University of Science and Technology, Hanoi, Vietnam; 4 Department of Agricultural Chemistry, College of Agriculture and Life Sciences, Chonnam National University, Gwangju, Republic of Korea; Tallinn University of Technology, ESTONIA

## Abstract

In the search for new antibacterial agents from natural sources, we revealed that a crude methanol extract of *Sapium baccatum* was highly active against *Ralstonia solanacearum*, a causal agent of a serious disease called bacterial wilt of tomato. The bioassay-guided fractionation of this extract resulted in the isolation of seven known active compounds, including gallic acid, methyl gallate, corilagin, tercatain, chebulagic acid, chebulinic acid, and quercetin 3-O-α-L-arabinopyranoside. Their chemical structures were determined by electrospray ionization mass spectrometry and nuclear magnetic resonance spectroscopy. An *in vitro* antibacterial bioassay using a broth microdilution method revealed that, except for quercetin 3-O-α-L-arabinopyranoside (MIC = 250 μg/mL), the isolated compounds exhibited strong antibacterial activity against *R*. *solanacearum* (MIC = 26–52 μg/mL). Among the seven compounds, methyl gallate exhibited the strongest broad-spectrum activity against most of the plant pathogenic bacteria tested (MIC = 26–250 μg/mL). In the *in vivo* experiments, the crude extract of *S*. *baccatum* at 2000 and 1000 μg/mL reduced the development of tomato bacterial wilt by 83 and 63%, respectively, under greenhouse conditions after 14 days of infection. The results suggested that the extracts of *S*. *baccatum* or isolated tannins could be used as natural bactericides for the control of bacterial wilt of tomato.

## Introduction

Phytopathogenic bacteria cause serious economic losses by reducing the yield of marketable quality crops [1]. *Ralstonia solanacearum*, which causes vascular wilt disease, is one of the most destructive pathogens [2]. This soil-born pathogen is found worldwide and has a large range of hosts, comprising more than 200 plant species, including tomato, potato, pepper, peanut, tobacco, and banana [2–4]. The direct yield losses caused by *R*. *solanacearum* differ widely according to the host, cultivar, climate, soil type, cropping pattern, and strain; the losses were up to 90% for tomato, potato, and banana [2].

The control of bacterial wilt has been considered difficult owing to the complex nature of the pathogen: endophytic growth patterns, survival in soil, transport in water, and a wide host range and biological diversity [2, 5]. The investigation into various methods for the control of bacterial wilt disease has spanned several decades. Yuliar [2] reported that studies of these methods conducted between 1984 and 2014 predominantly described the biological methods (54%), followed by cultural practices (21%), chemical methods (8%), and physical methods (6%). Plant disease control has been largely dependent on the use of chemical pesticides. Unfortunately, the use of chemical pesticides has been strongly limited owing to their association with environmental pollution, poisonous effects, and antibiotic resistance [2, 6, 7]. As the demand for environmentally acceptable pesticides is increasing, the development of safe and effective antimicrobial agents is essential for the treatment of plant disease.

Plants contain chemically diverse compounds that can be used directly as pesticides with reduced harmful effects [8]. Globally, the use of botanicals as alternatives to synthetic pesticides has increased annually. In particular, the growing demand for botanical pesticides has been much higher in developed or industrialized countries because of the increase in organic food production [9]. Botanicals have many advantages over synthetic chemicals, such as less or no residues on food because of rapid degradation, little or no harmful effect in humans or on the environment, and cost effectiveness [9]. However, botanical pesticides have some limitations, such as slow and lower efficacy compared with chemical pesticides and less efficacy when applied to fields; thus, the development of novel formulations with enhanced efficacy and longer shelf life is required [10–12]. Nowadays, nanotechnology has been applied in formulating, thus plant parts such as fruit, leaf, bark, seed, and stem extracts have been used for synthesis of nanoparticles, as effective formulations for the phytopathogens control [12]. There are many botanical pesticides such as plant extracts and their compounds possess antibacterial activities against phytopathogenic bacteria including *R*. *solanacearum* [12]. Most current studies that have focused on botanicals for the control of *R*. *solanacearum* utilized *in vitro* assays or potted plants [13–16]. To the best of our knowledge, there are no commercialized botanical products currently available for the control of bacterial wilt of tomato. Therefore, the search and development of highly active botanical pesticides for the control of tomato bacterial wilt are quite necessary.

In the search for antibacterial agents from Vietnamese plants, we found that the methanol extract of aerial parts of *Sapium baccatum* was highly active against *R*. *solanacearum*. *S*. *baccatum* is widespread across South Asia and has been used as a traditional medicine in Malaysia [17, 18]. Although several compounds, including bukittingine, lupeol, betulin, β-taraxerol, taraxerone, aleuritolic acid, 3-acetoxy-aleuritolic acid, 1-hexacosanol, β-sitosterol, stigmasterol, docosyl *trans*-isoferulate, and docosanoic acid 2',3'-dihydroxypropyl ester have been extracted from *S*. *baccatum* [17–19], limited information is available about the antimicrobial activities of these components or whether *S*. *baccatum* has additional active compounds. The aims of this study were: 1) to isolate and identify antibacterial compounds from *S*. *baccatum*; 2) to examine the antibacterial spectra of the isolated compounds against plant pathogenic bacteria; and 3) to evaluate the disease control efficacies of the methanol extract of *S*. *baccatum* on bacterial wilt of tomato under greenhouse conditions.

## Materials and methods

### Bacterial strains and culture conditions

The following strains of plant pathogenic bacteria were used for antibacterial activity assays: *Acidovorax avenae* subsp. *cattleyae* SL4351, the causal agent of bacterial brown spot in Phalaenopsis (*Phalaenopsis* sp.); *Agrobacterium tumefaciens* SL2434, the causal agent of crown gall in apple (*Malus domestica*); *Burkholderia glumae* SL4269, the causal agent of bacterial panicle blight in rice (*Oryza sativa*); *Clavibacter michiganensis* subsp. *michiganensis* SL4135, the causal agent of bacterial wilt and canker in tomato (*Solanum lycopersicum*); *Pectobacterium carotovorum* subsp. *carotovorum* SL290, the causal agent of bacterial soft rot in potato (*Solanum tuberosum*); *Pectobacterium chrysanthemi* SL3218, the causal agent of bacterial leaf rot in aloe (*Aloe vera*); *Pseudomonas syringae* pv. *actinidiae* CJW7, the causal agent of bacterial canker in kiwifruit (*Actinidia deliciosa*); *Pseudomonas syringae* pv. *lachrymans* SL308, the causal agent of cucumber angular leaf spot (*Cucumis sativus*); *R*. *solanacearum* SL1944, the causal agent of bacterial wilt in tomato (*S*. *lycopersicum*); and *Xanthomonas arboricola* pv. *pruni* SL4370, the causal agent of bacterial spot of peach (*Prunus persica*). All of these bacteria were isolated from the infected tissues by Dr SD Lee of the National Academy of Agricultural Sciences [4], except for *P*. *syringae* pv. *actinidiae* CJW7, which was isolated by Prof. YJ Koh of Sunchon National University [20] and *R*. *solanacearum*, which was isolated by Prof. SW Lee of Dong-A University [4, 21]. All strains were grown on tryptic soy agar (TSA; Becton, Dickinson and Co., Sparks, MD, USA) or tryptic soy broth (TSB). *P*. *syringae* pv. *actinidia*e and *X*. *arboricola* pv. *pruni* were grown at 25°C for 18–36 h and all other strains were cultured at 30°C for 18–36 h.

### Plant material

The aerial parts of *S*. *baccatum* were collected by the Department of Phytochemistry, Vietnam Institute of Industrial Chemistry (Hanoi, Vietnam). Plant species were identified by Dr The Bach Tran from the Institute of Ecology and Biological Resources (Hanoi, Vietnam) and voucher specimens were deposited in the laboratory.

### Extraction and isolation of antibacterial compounds

The dry powdered material of *S*. *baccatum* (200 g) was extracted twice with 90% methanol (2 × 3 L) for 48 h at room temperature. The extracts were filtered through Whatman No. 1 filter paper and the filtrates were concentrated by using a rotary evaporator under vacuum to yield a crude extract (24.5 g). A portion of the methanol extract (15 g) was suspended in 500 mL distilled water and then successively partitioned twice with *n*-hexane, ethyl acetate (EtOAc), and *n*-butanol (BuOH). Of the four layers, the EtOAc and BuOH layers showed strong antibacterial activity against *R*. *solanacearum* as determined by the broth microdilution method [4]. Therefore, further isolation of active compounds was conducted from these two layers.

The EtOAc layer (1.55 g) was successively eluted on a silica gel column (2.5 × 60.0 cm, Kiesel gel 60, 100 g, 230–400 mesh, E. Merck) with mixtures of dichloromethane (DCM)/methanol (MeOH) (90:10, v/v, 200 mL; 85:15, v/v, 200 mL; 80:20, v/v, 500 mL; 70:30, v/v, 500 mL), yielding five fractions, E1–E5. The fractions were monitored with thin-layer chromatography (TLC, Silica gel 60 F_254_, 0.25 mm layer thickness; E. Merck) with the developing solvent DCM/MeOH (85:15, v/v). Fraction E2 (233 mg) was separated on a Sephadex LH20 column (2.5 × 60.0 cm, 60 g, 70–100 μm, Sigma-Aldrich) via successive elution with mixtures of DCM/MeOH (9:1, v/v, 100 mL; 8:2, v/v, 200 mL; 7:3, v/v, 300 mL), to obtain seven fractions designated E21–E27. Fraction E25 (21 mg) containing compound **2** was successfully separated on a Sephadex LH20 column (1.0 × 30.0 cm, 10 g, 70–100 μm) by using DCM/MeOH (7:3, v/v, 100 mL). Compound **2** was further purified on a silica gel column (1.0 × 30.0 cm, Kiesel gel 60, 10 g, 230–400 mesh) and eluted with DCM/MeOH (9:1, v/v, 50 mL), yielding a pure compound (5 mg).

Fraction E3 (900 mg) was separated on a Sephadex column (3.0 × 60.0 cm, 100 g, 70–100 μm; Sigma-Aldrich) and successively eluted with DCM/MeOH (8:2, v/v, 200 mL; 7:3, v/v, 500 mL; 5:5, v/v, 500 mL; 3:7, v/v, 700 mL), which yielded 10 fractions designated E31–E310. The fractions were monitored by using reversed-phase TLC (Silica gel 60 RP-18 F_254_, 0.25-mm layer thickness; E. Merck) with the developing solvent MeOH/water (W) (4:6, v/v). To obtain compound **7** (4.3 mg), fraction E35 (40 mg) was separated with a LiChroprep RP-18 column (1.0 × 30.0 cm, 10 g, 40–63 μm; E. Merck) and eluted with MeOH/W (2:8, v/v; 3:7, v/v; 4:6, v/v, 50 mL of each).

Fraction E38 (120 mg) was first purified with a LiChroprep RP-18 column (1.5 × 30.0 cm, 15 g, 40–63 μm) eluted with a mixture of MeOH/W (2:8, v/v, 50 mL; 3:7, v/v, 100 mL; 4:6, v/v, 50 mL), further purified with the Sephadex LH20 column (1.0 × 30.0 cm, 10 g, 70–100 μm) and eluted with 100% MeOH to afford compound **1** (43 mg). Fraction E310 (250 mg) was also separated with the LiChroprep RP-18 column (1.5 × 30.0 cm, 15 g, 40–63 μm) and eluted with MeOH/W (2:8, v/v, 30 mL; 3:7, v/v, 30 mL; 4:6, v/v, 100 mL), yielding compound **5** (41 mg), compound **4** (5 mg), and compound **6** (5 mg).

The BuOH layer (7 g) was loaded on a silica gel column (6.0 × 60.0 cm, Kiesel gel 60, 480 g, 230–400 mesh) and successively eluted with DCM/MeOH/W (70:25:5, v/v/v, 1.5 L; 65:30:5, v/v/v, 1.5 L; 50:45:7, v/v/v, 1 L; 30:60:10, v/v/v, 1 L), which yielded nine fractions, B1–B9. Because the active fractions B6–B8 exhibited patterns similar to those of the main components based on TLC analysis with the developing solvent DCM/MeOH/W (70:25:5, v/v/v), they were pooled and further purified. A portion (600 mg) of the combined fraction (2.3 g) was first separated on a Sephadex LH20 column (2.5 × 60.0 cm, 100 g, 70–100 μm) and successively eluted with DCM/MeOH (3:7, v/v; 2:8, v/v; 1:9, v/v; 100% MeOH; 200 mL of each) and then further purified with a Sep-Pak C18 cartridge (Sep-Pak Vac 35cc, 10 g, Waters) and eluted with MeOH/W (1:9, 2:8, 3:7, 4:6, 5:5, v/v, 100 mL of each) to yield compound **3** (50 mg).

### Structure determination of antibacterial compounds

The chemical structures of the active components were determined by using mass spectrometry, nuclear magnetic resonance (NMR) spectroscopy, and comparison with values reported in the literature. The electrospray ionization mass spectra (ESI-MS) of the isolated compounds were recorded on an MSD1100 single-quadruple mass spectrometer equipped with an electrospray ionizer (Hewlett-Packard Co., Palo Alto, CA, USA). ^1^H and ^13^C NMR spectra were measured using methanol-d_4_, dimethyl sulfoxide (DMSO)-d_6_ or acetone-d_6_ (E. Merck) with a Bruker AMX-500 spectrometer (Bruker, Analytische Messtechnik Gmbh, Rheinstetten, Germany). Chemical shifts were calculated using tetramethylsilane as the internal standard.

### *In vitro* antibacterial activity

The antibacterial activities of the isolated compounds (compounds **1**–**7**) were measured with the broth microdilution method against various plant pathogenic bacteria, including *R*. *solanacearum*. Briefly, suspensions of plant pathogenic bacteria in TSB media (100 μL) with inocula of 10^6^ CFU/mL were added to the wells of sterile 96-well plates. The stock solutions of the isolated compounds (25 mg/mL) were diluted 100-fold in the first wells and then subjected to two-fold serial dilutions in the growth media. The final concentrations of the isolated compounds were in the range 7.8–250 μg/mL. DMSO (1%), which corresponded to the highest concentration and did not affect the bacterial growth, was used as the negative control. Streptomycin sulfate was used as the positive control.

The inoculated plates were incubated at 30°C (except for *P*. *syringae* pv. *actinidiae* and *X*. *arboricola* pv. *pruni*, which were incubated at 25°C) for 18–36 h after shaking at 300 rpm for 10 min on a microplate shaker. The minimum inhibitory concentration (MIC) was defined as the lowest concentration that completely inhibited the growth of the bacteria. The assay was performed three times with three replicates for each extract at all concentrations tested.

### Disease control efficacy of the methanol extract of *S*. *baccatum* against bacterial wilt of tomato

To evaluate the efficacy of the methanol extract of *S*. *baccatum* in controlling tomato bacterial wilt caused by *R*. *solanacearum* SL1944 (race 1, biovar 4) [4, 21], we used 3-week-old ‘Seokwang’ tomato plants at the four- to five-true-leaf stage. The plants were grown in vinyl pots with a volume of 90 mL in a greenhouse and were then transplanted into vinyl pots with a volume of 180 mL (one plant per pot). Two different amounts of starting material (100 and 200 mg) of the methanol plant extract were dissolved in 2 mL MeOH and then diluted in 98 mL distilled water containing 250 μg/mL Tween-20 to obtain test concentrations of 1000 and 2000 μg/mL. *R*. *solanacearum* was grown in TSA Petri dishes at 30°C for 48 h, harvested with distilled water, and adjusted to an optical density at 600 nm of 0.1 (approximately 1.5 × 10^8^ CFU/mL). The two methanol extract solutions were applied to the soil of each pot (20 mL per pot). After 3 h of treatment, a cell suspension (20 mL) of *R*. *solanacearum* was inoculated into the soil of each pot. Streptomycin sulfate (200 μg/mL) was used as the positive control and distilled water solutions containing Tween-20 (250 μg/mL) and MeOH (2%) were used as negative controls. The plants were maintained in a controlled climate at 30 ± 2°C and a relative humidity of 70–80%. The pots were arranged as a randomized complete block with five replicates per treatment. The experiment was repeated three times [4, 22].

The disease severity was ranked daily for 14 days and recorded on a scale of 0–4 as described by He et al. (1983): 0, no symptoms; 1, one leaf wilted; 2, two or three leaves wilted; 3, four or more leaves wilted; and 4, plant dead [4, 23]. The control value was calculated by using following formula [22]:

Control value (%)=100×(disease severity of control – disease severity of treatment)/disease severity of control

### Statistical analysis

The data were assessed by one-way analysis of variance (ANOVA) and the significance of the treatments was determined by Tukey’s honest significant difference (HSD) for multiple comparisons (*p* = 0.05). Statistical analyses were performed by using SAS software (version 12.0, SAS Institute, Cary, NC). Differences were considered statistically significant for *p* values less than 0.05.

## Results and discussion

### Structure determination of antibacterial compounds

**Fig 1:**
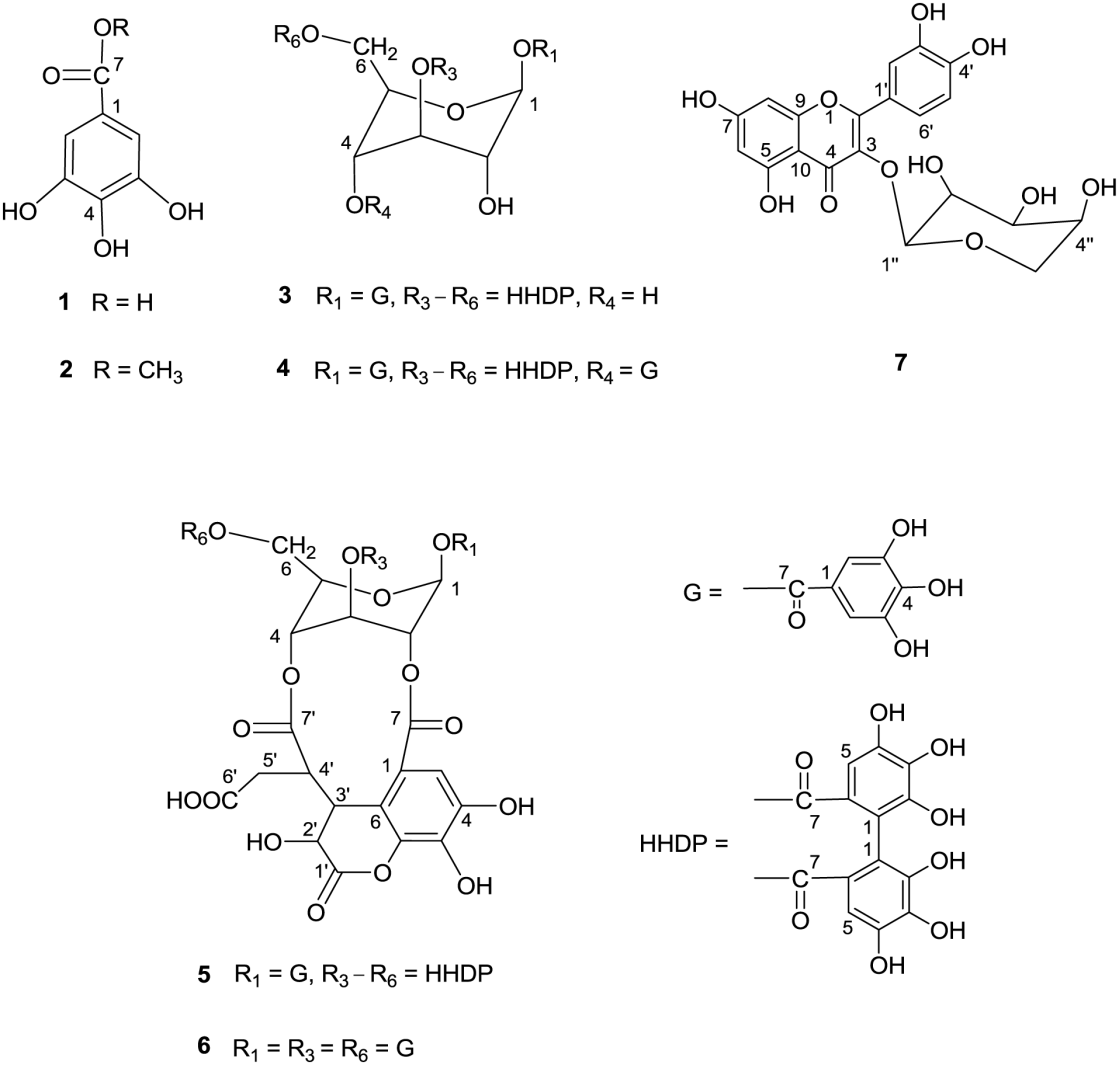
Chemical structures of seven antibacterial compounds isolated from the aerial parts of *Sapium baccatum*. Compound **1**, gallic acid; compound **2**, methyl gallate; compound **3**, corilagin; compound **4**, tercatain; compound **5**, chebulagic acid; compound **6**, chebulinic acid; compound **7**, quercetin 3-O-α-L-arabinopyranoside.

The bioassay-guided fractionation of *S*. *baccatum* crude extract (15 g) led to the isolation of seven compounds. The chemical structures of these compounds were determined based on ^1^H NMR, ^13^C NMR, and ESI-MS data and through comparison with the previously reported literature values. The compounds were identified as gallic acid (compound **1**) [24–26], methyl gallate (compound **2**) [24–26], corilagin (compound **3**) [25–27], tercatain (compound **4**) [28, 29], chebulagic acid (compound **5**) [25, 26, 30, 31], chebulinic acid (compound **6**) [26, 30, 31], and quercetin 3-O-α-L-arabinopyranoside or guaijaverin (compound **7**) [32–34] (Fig 1). The ^1^H, ^13^C NMR, and ESI-MS data of these compounds are shown in S1–S5 Tables.

These compounds have previously been isolated from plant extracts, most of them belong to tannins group which possess various bioactivities including antibacterial activity [35]: gallic acid and methyl gallate from extracts of *Sedum takesimense* aerial parts [4]; *Euphorbia helioscopia* whole plants [24]; *Dimocarpus longan* seeds [25] and *Terminalia* spp. fruits, including *T*. *bellerica*, *T*. *chebula*, and *T*. *horrida* [26]; corilagin from extracts of the *D*. *longan* seeds [25] and *Terminalia* spp. fruits [26], *Punica granatum* leaves [27], and *E*. *fischeriana* roots [29]; tercatain from extracts of leaves of *T*. *catappa* [28] and *E*. *fischeriana* roots [29]; chebulagic and chebulinic acids from extracts of the *D*. *longan* seeds and the *Terminalia* spp. fruits [25, 26, 30, 31]; and quercetin 3-O-α-L-arabinopyranoside from extracts of *Woodfordia fruticosa* [32] and *Psidium guajava* leaves [36], and *Vaccinium macrocarpon* powder [34]. To the best of our knowledge, we have reported the first isolation of these seven compounds from *S*. *baccatum*.

### *In vitro* antibacterial activity

The antibacterial activity of the compounds isolated from *S*. *baccatum* is presented in Table 1. Among the seven compounds, methyl gallate (compound **2**) exhibited the strongest broad-spectrum activity against most of the plant pathogenic bacteria tested, with MIC values between 26.0 and 250 μg/mL (except against *A*. *avenae* subsp. *cattleyae*). Of the glucoside gallates (compounds **3**–**6**), corilagin (compound **3**) and chebulagic acid (compound **5**) showed a slightly stronger activity than that of tercatain (compound **4**) and chebulinic acid (compound **6**), respectively. Gallic acid was less active than other hydrolysable tannins (compounds **2**–**6**) and quercetin 3-O-α-L-arabinopyranoside (compound **7**) was the least active compound (Table 1).

**Table 1:** Minimum inhibitory concentration (MIC) values of isolated compounds against plant pathogenic bacteria.

Bacterium	MIC (μg/mL)						
	1	2	3	4	5	6	7
***Acidovorax avenae* subsp. *cattleyae***	104.2 ± 31.3 b	>250	104.2 ± 31.3 b	250 a	208.3 ± 62.5 a	>250	>250
***Agrobacterium tumefaciens***	>250	250	>250	>250	>250	>250	>250
***Burkholderia glumae***	>250	62.5 c	104.2 ± 31.3 c	208.3 ± 62.5 ab	166.7 ± 62.5 b	250 a	>250
***Clavibacter michiganensis* subsp. *michiganensis***	>250	88.3 ± 31.3	>250	>250	>250	>250	>250
***Pectobacterium carotovorum* subsp. *carotovorum***	>250	250	>250	>250	>250	>250	>250
***Pectobacterium chrysanthemi***	>250	104.2 ± 31.3	>250	>250	>250	>250	>250
***Pseudomonas syringae* pv. *actinidiae***	>250	166.7 ± 62.5 b	250 a	166.7 ± 62.5 b	104.2 ± 31.3 c	104.2 ± 31.3 c	>250
***Pseudomonas syringae* pv. *lachrymans***	>250	208.3 ± 62.5	>250	>250	>250	>250	>250
***Ralstonia solanacearum***	41.7 ± 15.6 bc	26.0 ± 7.8 c	31.3 bc	52.1 ± 15.6 b	52.1 ± 15.6 b	52.1 ± 15.6 b	250 a
***Xanthomonas arboricola* pv. *pruni***	>250	62.5 a	88.3 ± 31.3 a	88.3 ± 31.3 a	52.1 ± 15.6 a	52.1 ± 15.6 a	>250

Compound **1**, gallic acid; compound **2**, methyl gallate; compound **3**, corilagin; compound **4**, tercatain; compound **5**, chebulagic acid; compound **6**, chebulinic acid; compound **7**, quercetin 3-O-α-L-arabinopyranoside.

Means within the same row followed by the same letter are not significantly different (*p* = 0.05) as determined by Tukey’s HSD test.

Of plant pathogenic bacteria tested, *R*. *solanacearum* was the most susceptible to all the isolated compounds, followed by *X*. *arboricola* pv. *pruni*, *P*. *syringae* pv. *actinidiae*, *B*. *glume* and *A*. *avenae* subsp. *cattleyae*. With the exception of compound **7** (MIC = 250 μg/mL), the isolated compounds showed impressive antibacterial activity against *R*. *solanacearum*, with very low MICs (26.0–52.1 μg/mL). Compounds **2**–**6** also exhibited strong antibacterial activities against *X*. *arboricola* pv. *pruni* (MIC = 52.1–88.3 μg/mL) (Table 1).

Methyl gallate (compound **2**) was much more active than gallic acid (compound **1**). The measurements of antibacterial activity in our study agreed with those of previous reports [4, 37, 38]. Compounds with similar structures, such as **3** and **4**, and **5** and **6**, had similar antibacterial activity and spectra (Table 1). Corilagin (compound **3**) has been reported against *Acinetobacter baumannii* [39], methicillin-resistant *Staphylococcus aureus* [40, 41], and *Escherichia coli* [41]. Chebulagic acid (compound **5**) and chebulinic acid (compound **6**) showed moderate antibacterial activity against *A*. *baumannii* [39]. The antibacterial activity of quercetin-3-O-α-L- arabinopyranoside or guaijaverin (compound **7**) against *Streptococcus mutans* has been reported [36]. To the best of our knowledge, information on the antibacterial activities of tercatain (compound **4**) is unavailable. In addition, this is the first report of the antibacterial activities of the isolated compounds (with the exception of compounds **1** and **2**) against plant pathogenic bacteria [4, 42].

### Effect of the methanol extract on tomato bacterial wilt

**Fig 2:**
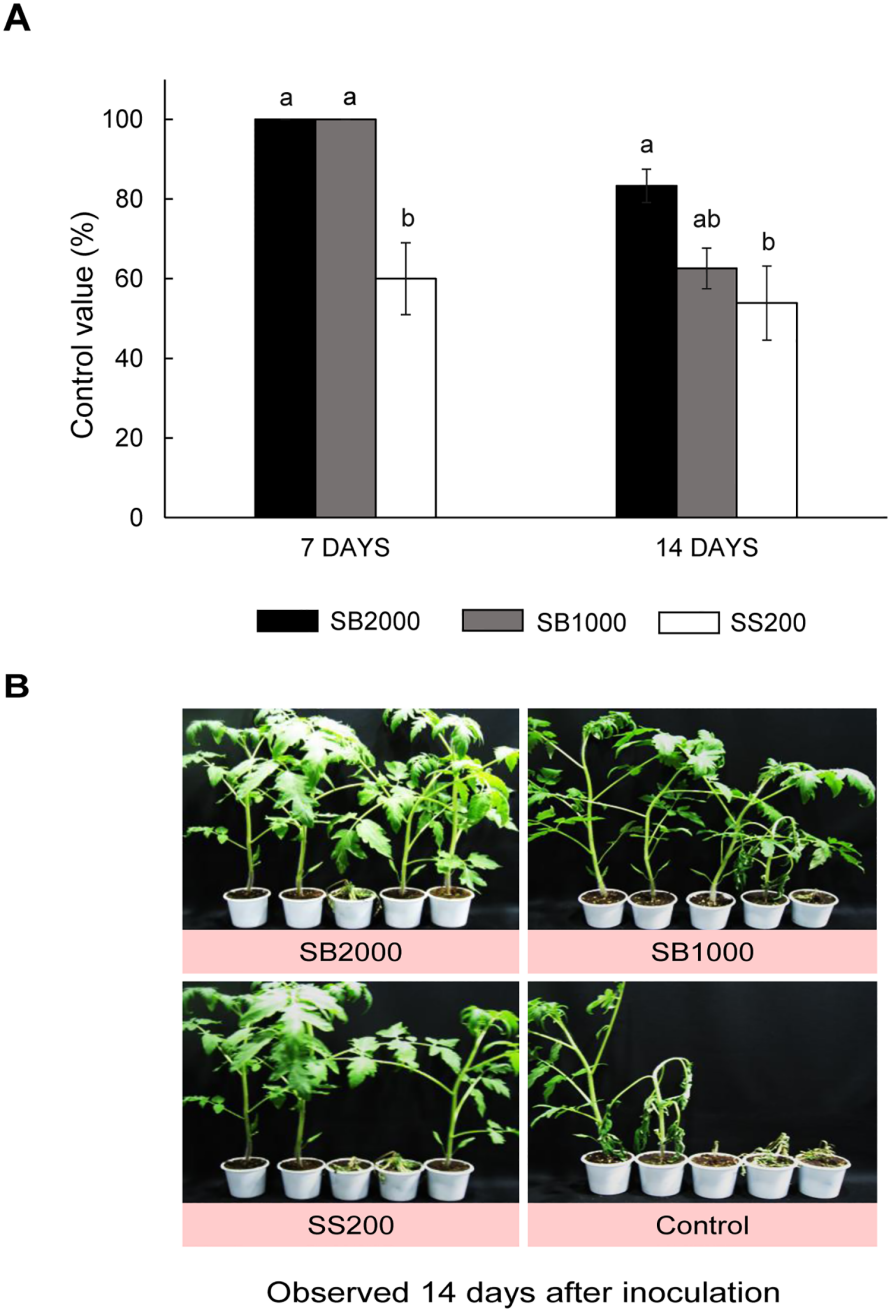
Effect of the methanol extract of *Sapium baccatum* on tomato bacterial wilt under greenhouse conditions (A) and the treated plants 14 days after inoculation (B). SB1000 and SB2000, 1000 and 2000 μg/mL methanol extract of *S*. *baccatum*, respectively; SS200, 200 μg/mL streptomycin sulfate. Each value represents the mean ± standard deviation of three experiments with five replicates. Means with the same number days after inoculation followed by the same letter above the bars are not significantly different (*p* = 0.05) as determined by Tukey’s HSD test.

In the *in vivo* experiment, wilt symptoms were observed 5 days after inoculation. The extracts efficiently suppressed the development of tomato bacterial wilt in a dose-dependent manner. At concentrations of 1000 and 2000 μg/mL, the methanol extract showed control efficacies of 100 and 100% after 7 days of inoculation, and 63 and 83% after 14 days of inoculation, respectively. The disease control efficacies of the extract at 1000 μg/mL were higher than those of 200 μg/mL streptomycin sulfate after 7 and 14 days of inoculation (Fig 2 and S6 Table). No phytotoxic symptoms appeared on the treated plants.

Recent studies revealed that several plant-derived products also exhibited potential antibacterial activity against *R*. *solanacearum* in *in vivo* tests. *Allium fistulosum* extract at concentrations of 50 and 100% significantly reduced the incidence of bacterial wilt of tomato: only 6 and 14% of the plants were affected, respectively, whereas the disease affected 61% of the plants in the untreated control [43]. The leaf extract of *Eichhorina crassipes* reduced the severity index of the bacterial wilt by more than 91% [44]. Several essential oils, such as cinnamon and clove oils [13], lemongrass and palmarosa oils and their components such as thymol [45, 46], were found to effectively reduce the *R*. *solanacearum* populations and incidence of bacterial wilt of tomato grown in infested soil. Methyl gallate at a concentration of 500 μg/mL showed a control efficacy of 65.2% in greenhouse conditions [42]. It is difficult to compare the results of our study with those of previous studies owing to a number of factors, such as different experimental design conditions, plants species, and bacterial species. In this study, the control efficacy of *S*. *baccatum* at a concentration of 2000 μg/mL was similar to that of the wettable powder formulation of the ethyl acetate layer of *S*. *takesimense* at a 200-fold dilution, as described in our previous study [4].

## Conclusions

In this study, seven antibacterial compounds were isolated from the methanol extracts of *S*. *baccatum*. The compounds showed potent *in vitro* antibacterial activities against *R*. *solanacearum*, except for quercetin 3-O-α-L-arabinopyranoside. In addition, most compounds exhibited strong antibacterial activities against *X*. *arboricola* pv. *pruni* and *B*. *glumae*. Methyl gallate and corilagin showed the strongest activities. These results suggested that the extracts from *S*. *baccatum* or their isolated compounds were promising antibacterial agents for the control of bacterial wilt of tomato.

Botanical pesticides are a promising alternative to reduce the harmful effects caused by use of synthetic pesticides. They have become more attractive after the increase in demand for organic food. Further research into production, formulation, and delivery may greatly assist and promote the development of botanical pesticides. Therefore, further studies are necessary to examine the toxicity of the *S*. *baccatum* extract, evaluate the disease control efficacy of the extract in various fields, and develop optimum formulations of the crude extracts for the control of tomato bacterial wilt.

## Supporting information

S1 Table NMR data of gallic acid and methyl gallate isolated from *Sapium baccatum* in methanol-d_4_. (DOCX)

S2 Table NMR data of corilagin and tercatain isolated from *Sapium baccatum* in DMSO-d_6_ and acetone-d_6_, respectively. (DOCX)

S3 Table NMR data of chebulagic acid and chebulinic acid isolated from *Sapium baccatum* in acetone-d_6_. (DOCX)

S4 Table NMR data of quercetin 3-O-α-L-arabinopyranoside isolated from *Sapium baccatum* in methanol-d_4_. (DOCX)

S5 Table ESI-MS data of seven active compounds isolated from *Sapium baccatum*. (DOCX)

S6 Table Effect of the methanol extract of *Sapium baccatum* on the control of tomato bacterial wilt under greenhouse conditions. (DOCX)
