# Supplementary material for: Antibacterial activity of tannins isolated from Sapium baccatum extract and use for control of tomato bacterial wilt
Source: PLoS One. 2017 Jul 25;12(7):e0181499. doi: 10.1371/journal.pone.0181499 (PMC5526539; doi:10.1371/journal.pone.0181499)
Supplement: S1 Table — (DOCX) [file pone.0181499.s001.docx]

S1 Table. NMR data of gallic acid and methyl gallate isolated from *Sapium baccatum* in methanol-d_4_.

|  | **Gallic acid** | |  | **Methyl gallate** | |
| --- | --- | --- | --- | --- | --- |
| **Position** | **^1^H** | **^13^C** |  | **^1^H** | **^13^C** |
| 1 |  | 121.99 |  |  | 121.26 |
| 2 | 7.07, s | 110.23 |  | 7.03, s | 109.81 |
| 3 |  | 146.35 |  |  | 146.48 |
| 4 |  | 139.53 |  |  | 139.79 |
| 5 |  | 146.35 |  |  | 146.48 |
| 6 | 7.07, s | 110.23 |  | 7.03, s | 109.81 |
| 7 |  | 170.55 |  |  | 169.00 |
| -OCH_3_ |  |  |  | 3.80 (3H, s) | 52.35 |
