# Supplementary material for: Antibacterial activity of tannins isolated from Sapium baccatum extract and use for control of tomato bacterial wilt
Source: PLoS One. 2017 Jul 25;12(7):e0181499. doi: 10.1371/journal.pone.0181499 (PMC5526539; doi:10.1371/journal.pone.0181499)
Supplement: S2 Table — (DOCX) [file pone.0181499.s002.docx]

S2 Table. NMR data of corilagin and tercatain isolated from *Sapium baccatum* in DMSO-d_6_ and acetone-d_6_, respectively

|  | | **Corilagin** | |  | **Tercatain** | |
| --- | --- | --- | --- | --- | --- | --- |
| **Position** | | **^1^H** | **^13^C** |  | **^1^H** | **^13^C** |
| Glucose | 1 | 6.21, d (7.3) | 92.64 |  | 6.27, d (4.0) | 93.78 |
|  | 2 | 3.88, brs | 72.10 |  | 4.29, d (4.7) | 69.66 |
|  | 3 | 4.59, brs | 78.01 |  | 4.98, brs | 72.15 |
|  | 4 | 4.22, brs | 64.41 |  | 5.76, d (3.4) | 64.05 |
|  | 5 | 4.36, t (8.0) | 76.81 |  | 4.56, t (7.8) | 74.24 |
|  | 6 | 4.24, dd (7.7, 11.0)  3.94, t (10.2) | 62.61 |  | 4.64, dd (7.8, 11.2)  4.36, dd (7.6, 11.1) | 63.85 |
| Galloyl  (R_1_) | 1 |  | 119.16 |  |  | 119.73 |
|  | 2 | 7.02, s | 109.44 |  | 7.20, s | 109.52 |
|  | 3 |  | 146.04 |  |  | 145.22 |
|  | 4 |  | 139.44 |  |  | 138.90 |
|  | 5 |  | 146.04 |  |  | 145.22 |
|  | 6 | 7.02, s | 109.44 |  | 7.20, s | 109.52 |
|  | 7 |  | 165.27 |  |  | 165.17 |
| Galloyl  (R_4_) | 1 |  |  |  |  | 119.42 |
|  | 2 |  |  |  | 7.18, s | 109.35 |
|  | 3 |  |  |  |  | 145.18 |
|  | 4 |  |  |  |  | 138.62 |
|  | 5 |  |  |  |  | 145.18 |
|  | 6 |  |  |  | 7.18, s | 109.35 |
|  | 7 |  |  |  |  | 165.07 |
| HHDP  (R_3_-R_6_) | 1 |  | 115.97, 116.25 |  |  | 114.90, 115.76 |
|  | 2 |  | 144.42, 144.75 |  |  | 144.03, 144.15 |
|  | 3 |  | 135.85, 135.98 |  |  | 135.76, 136.19 |
|  | 4 |  | 145.20, 145.28 |  |  | 144.15, 144.46 |
|  | 5 | 6.50, 6.56, s | 106.51, 107.39 |  | 6.75, 6.89, s | 108.78, 108.78 |
|  | 6 |  | 123.52, 124.54 |  |  | 124.28, 124.39 |
|  | 7 |  | 167.18, 167.54 |  |  | 166.40, 167.93 |
