# Supplementary material for: Antibacterial activity of tannins isolated from Sapium baccatum extract and use for control of tomato bacterial wilt
Source: PLoS One. 2017 Jul 25;12(7):e0181499. doi: 10.1371/journal.pone.0181499 (PMC5526539; doi:10.1371/journal.pone.0181499)
Supplement: S3 Table — (DOCX) [file pone.0181499.s003.docx]

S3 Table. NMR data of chebulagic acid and chebulinic acid isolated from *Sapium baccatum* in acetone-d_6_.

|  | | **Chebulagic acid** | |  | **Chebulinic acid** | |
| --- | --- | --- | --- | --- | --- | --- |
| **Position** | | **^1^H** | **^13^C** |  | **^1^H** | **^13^C** |
| Glucose | 1 | 6.51, brs | 91.61 |  | 6.52, brs | 91.58 |
|  | 2 | 5.52, brs | 70.70 |  | 5.47, brs | 70.47 |
|  | 3 | 5.95, brs | 61.73 |  | 6.35, brs | 61.47 |
|  | 4 | 5.23, d (3.6) | 66.26 |  | 5.08, m | 68.21 |
|  | 5 | 4.80, d (7.5) | 73.64 |  | 4.75, m | 75.04 |
|  | 6 | 4.41, dd (7.2, 10.0)  4.78, m | 63.87 |  | 4.83, m  4.71, m | 63.97 |
| Galloyl  (R_1_) | 1 |  | 119.62 |  |  | 119.63 |
|  | 2 | 7.19, s | 110.35 |  | 7.28, s | 109.41 |
|  | 3 |  | 145.85 |  |  | 145.26 |
|  | 4 |  | 139.93 |  |  | 139.22 |
|  | 5 |  | 145.85 |  |  | 145.26 |
|  | 6 | 7.19, s | 110.35 |  | 7.28, s | 109.41 |
|  | 7 |  | 168.68 |  |  | 164.57 |
| HHDP (R_3_-R_6_)  /Galloyls  (R_3_, R_6_) | 1 |  | 115.12, 115.62 |  | 7.05, 7.22, s | 109.50, 109.03 |
|  | 2 |  | 144.47, 145.23 |  |  | 145.36, 145.16 |
|  | 3 |  | 136.14, 137.55 |  |  | 140.22, 138.24 |
|  | 4 |  | 144.81, 145.05 |  |  | 145.36, 145.16 |
|  | 5 | 6.66, 7.09, s | 107.43, 110.06 |  | 7.05, 7.22, s | 109.50, 109.03 |
|  | 6 |  | 124.19, 125.16 |  |  | 118.72, 118.80 |
|  | 7 |  | 165.57, 165.27 |  |  | 164.42, 164.66 |
| Chebulloyl | 1 |  | 118.31 |  |  | 117.58 |
|  | 2 | 7.53, s | 117.11 |  | 7.55, s | 116.17 |
|  | 3 |  | 146.50 |  |  | 145.87 |
|  | 4 |  | 139.69 |  |  | 138.52 |
|  | 5 |  | 140.78 |  |  | 140.22 |
|  | 6 |  | 116.79 |  |  | 115.23 |
|  | 7 |  | 166.33 |  |  | 166.14 |
|  | 1’ |  | 169.77 |  |  | 169.15 |
|  | 2’ | 4.96, d (7.2) | 66.15 |  | 4.97, d (7.2) | 65.75 |
|  | 3’ | 5.12, dd (1.4, 7.2) | 40.90 |  | 5.18, dd (1.6, 7.2) | 40.31 |
|  | 4’ | 3.88, t (7.7) | 39.21 |  | 3.96, m | 38.83 |
|  | 5’ | 2.20, d (7.7), 2H | 30.08 |  | 2.26, d (7.7), 2H | 29.58 |
|  | 6’ |  | 173.11 |  |  | 173.05 |
|  | 7’ |  | 173.57 |  |  | 173.16 |
