# Supplementary material for: Antibacterial activity of tannins isolated from Sapium baccatum extract and use for control of tomato bacterial wilt
Source: PLoS One. 2017 Jul 25;12(7):e0181499. doi: 10.1371/journal.pone.0181499 (PMC5526539; doi:10.1371/journal.pone.0181499)
Supplement: S4 Table — (DOCX) [file pone.0181499.s004.docx]

**S4 Table. NMR data of quercetin 3-O-α-L-arabinopyranoside isolated from *Sapium baccatum* in methanol-d_4_.**

|  | **Quercetin 3-O-**α-L**-arabinopyranoside** | |
| --- | --- | --- |
| **Position** | **^1^H** | **^13^C** |
| 2 |  | 158.67 |
| 3 |  | 135.62 |
| 4 |  | 179.47 |
| 5 |  | 163.07 |
| 6 | 6.23, d (2.1) | 99.88 |
| 7 |  | 166.13 |
| 8 | 6.42, d (2.1) | 94.69 |
| 9 |  | 158.43 |
| 10 |  | 105.60 |
| 1’ |  | 122.86 |
| 2’ | 7.77, d (2.2) | 117.42 |
| 3’ |  | 145.99 |
| 4’ |  | 149.97 |
| 5’ | 6.89, d (8.4) | 116.16 |
| 6’ | 7.60, dd (8.4, 2.2) | 123.01 |
| α-L-arabinopyranose |  |  |
| 1’’ | 5.19, d (6.6) | 104.61 |
| 2’’ | 3.92, dd (8.5, 6.6) | 72.87 |
| 3’’ | 3.66, dd (8.5, 3.2) | 74.12 |
| 4’’ | 3.83, (m) | 69.15 |
| 5’’ | 3.84, dd (13.7, 3.6)  3.47, dd (13.7, 3.3) | 66.99 |
