# Supplementary material for: Antibacterial activity of tannins isolated from Sapium baccatum extract and use for control of tomato bacterial wilt
Source: PLoS One. 2017 Jul 25;12(7):e0181499. doi: 10.1371/journal.pone.0181499 (PMC5526539; doi:10.1371/journal.pone.0181499)
Supplement: S5 Table — (DOCX) [file pone.0181499.s005.docx]

**S5 Table. ESI-MS data of seven active compounds isolated from *Sapium baccatum***

| No. | Compound | Molecular formula | [M-H]^−^ *m/z* | Major fragment ions: *m/z* |
| --- | --- | --- | --- | --- |
| 1 | Gallic acid | C_7_H_6_O_5_ | 169 | 125 |
| 2 | Methyl gallate | C_8_H_8_O_5_ | 183 | 124 |
| 3 | Corilagin | C_27_H_22_O_18_ | 633 | n.d. |
| 4 | Tercatain | C_34_H_26_O_22_ | 785 | n.d. |
| 5 | Chebulagic acid | C_41_H_30_O_27_ | 953 | n.d. |
| 6 | Chebulinic acid | C_41_H_32_O_27_ | 955 | n.d. |
| 7 | Quercetin 3-O-α-L-arabinopyranoside | C_20_H_18_O_11_ | 433 | 301 |

n.d., not detected.
