# Supplementary material for: Antibacterial activity of tannins isolated from Sapium baccatum extract and use for control of tomato bacterial wilt
Source: PLoS One. 2017 Jul 25;12(7):e0181499. doi: 10.1371/journal.pone.0181499 (PMC5526539; doi:10.1371/journal.pone.0181499)
Supplement: S6 Table — (DOCX) [file pone.0181499.s006.docx]

S6 Table. Effect of the methanol extract of *Sapium baccatum* on the control of tomato bacterial wilt under greenhouse conditions.

|  | **Control value (%)** | |
| --- | --- | --- |
| **Treatment** | **7 days** | **14 days** |
| SB2000 | 100 a | 83.3 ± 4.2 a |
| SB1000 | 100 a | 62.6 ± 5.1 ab |
| SS200 | 60.0 ± 9.0 b | 53.9 ± 9.3 b |

Control value was calculated 7 and 14 days after inoculation using the following formula: Control value (%) = 100 × (disease severity of control – disease severity of treatment) / disease severity of control; means ± standard deviation of three run with five replicates; means within the same column followed by the same letter are not significantly different (*p* = 0.05) in a Tukey’s HSD test. SB1000 and SB2000, 1000 and 2000 µg/mL methanol extract of *S. baccatum*, respectively; SS200, 200 µg/mL streptomycin sulfate.
